# Application of pristine and doped SnO_2_ nanoparticles as a matrix for agro-hazardous material (organophosphate) detection

**DOI:** 10.1038/srep42510

**Published:** 2017-02-14

**Authors:** Naushad Khan, Taimur Athar, H. Fouad, Ahmad Umar, Z. A. Ansari, S. G. Ansari

**Affiliations:** 1 Centre for Interdisciplinary Research in Basic Sciences, Jamia Millia Islamia, New Delhi, 110025 India; 2 Indian Institute of Chemical Technology, Telangana, Hyderabad 50007, India; 3 Department of Applied Medical Science, Riyadh Community College, King Saud University, Riyadh, 11437 Saudi Arabia; 4 Biomedical Engineering Department, Faculty of Engineering, Helwan University, P.O. Box, 11792, Helwan, Egypt; 5 Promising Centre for Sensors and Electronic Devices, Najran University, P.O. Box 1988, Najran, 11001, Saudi Arabia; 6 Department of Chemistry, Faculty of Sciences and Arts, Najran University, P.O. Box 1988, Najran, 11001, Saudi Arabia

## Abstract

With an increasing focus on applied research, series of single/composite materials are being investigated for device development to detect several hazardous, dangerous, and toxic molecules. Here, we report a preliminary attempt of an electrochemical sensor fabricated using pristine Ni and Cr–doped nano tin oxide material (SnO_2_) as a tool to detect agro-hazardous material, i.e. Organophosphate (OP, chlorpyrifos). The nanomaterial was synthesized using the solution method. Nickel and chromium were used as dopant during synthesis. The synthesized material was calcined at 1000 °C and characterized for morphological, structural, and elemental analysis that showed the formation of agglomerated nanosized particles of crystalline nature. Screen-printed films of powder obtained were used as a matrix for working electrodes in a cyclic voltammogram (CV) at various concentrations of organophosphates (0.01 to 100 ppm). The CV curves were obtained before and after the immobilization of acetylcholinesterase (AChE) on the nanomaterial matrix. An interference study was also conducted with hydroquinone to ascertain the selectivity. The preliminary study indicated that such material can be used as suitable matrix for a device that can easily detect OP to a level of 10 ppb and thus contributes to progress in terms of desired device technology for the food and agricultural-industries.

With an increasing focus on applied research, researchers are using a series of materials, including composite, doped, and undoped metal oxides, for the development of devices to detect several hazardous, dangerous, and toxic molecules either directly or indirectly in the air or a solution. This has resulted in a large number of publications mainly from an academic perspective, with some reports on device development. The key part for such device development freezes on the choice and synthesis of tailored materials with desirable properties. Many sectors related to human comfort have taken a tremendous lead in the quick and early detection of such molecules. For example, medical diagnosis requires device-based investigation kits/tools for quick analysis, unlike usual lab techniques, and few devices are on the market at this time. Similarly, the agriculture industry also requires technology that can be helpful to farmers for checking soil/fertilizer quality and crops by monitoring use of insecticide/pesticides (organophosphate). This industry needs a device that is portable and can be carried to the farms/fields with ease of operation. In modern agriculture, pesticides/insecticides are being extensively used to produce high-yield crops with no concern for the amount of toxicity involved and that leads to several neurological diseases^1^,^2^,^3^,^4^,^5^,^6^,^7^,^8^,^9^.

Traditionally, instruments are available to detect pollutants through gas chromatography (GC), electrophoresis, thin layer chromatography (TLC), mass spectrophotometry (MS), and high-performance liquid chromatography (HPLC). At the same time, these instruments require a skilled operator and a long operation time, and the operation costs are high^10^,^11^,^12^,^13^,^14^. In recent times, OPs (pesticides) are used in comparatively larger amounts than organochlorine pesticides, which pose a greater risk in terms of toxicity to the environment because they inhibit acetyl cholinesterase (AChE, EC3.1.1.7), leading to pesticide residue in fruits/food as well as in the environment, requiring an immediate solution. Therefore, a form of device that can quickly detect OP at the lowest possible concentrations is needed immediately. The development of such a device would require a suitable material that can be used as a matrix/transducer for signal conversion and reliable/reproducible detection.

Nanomaterials of metal oxides can be suitable candidates for such device fabrication, as they offer higher surface-to-volume ratio and the tailored electronic properties that are required for low power operation. Tin oxide is a well-studied non-stoichiometric compound used for gas sensing, biosensing, and optical devices can also be used for OP sensing in its pristine and doped forms to optimize its sensing characteristics^15^,^16^.

For example, Ni-doped SnO_2_ nanoparticles with 0–5 wt% Ni were synthesized by Lavanya N *et al*. using the microwave irradiation method with Horse Radish Peroxidase (HRP) for H_2_O_2_ sensing by forming a film^17^. In another report, Singkammo S *et al*. used flame spray to synthesize SnO_2_ nanoparticles by doping with 0.1 to 2 wt% Ni and 0.1 to 5 wt% graphene for acetone sensing by depositing thick films of approximately 12 to 18 μm thickness by spin-coating on Au/Al_2_O_3_ substrates^18^.

K. Subramanyama *et al*. used pure SnO_2_ and 1, 3, 5 and 7 at% of Cr-doped SnO_2_, synthesized by the chemical co-precipitation method to understand the effects of Cr on the properties of SnO_2_ nanoparticles^19^. In another report, Lavanya N *et al*. synthesized Cr-doped SnO_2_ nanoparticles using microwave and fabricated a biosensor to test riboflavin (RF). Cr concentrations was varied from 0 to 5 wt%^20^. Miguel García-Tecedor *et al*. recently fabricated Cr-doped SnO_2_ microtubes by thermal evaporation and found homogeneously distributed Cr along the tubes at a concentration of approximately 1 at%. They studied the optical properties of the microtubes that showed a purple color in contrast to the undoped transparent microtubes^21^. However, reports on OP sensing using doped SnO_2_ are rare.

Therefore, in this study, Ni and Cr–doped SnO_2_ was synthesized with an aim to develop an electrochemical sensor to detect OP in the solution. The sensor’s characteristics were obtained in terms of redox current, scan rate studies for understanding charge transfer characteristics, and interference studies.

## Results

**Figure 1:**
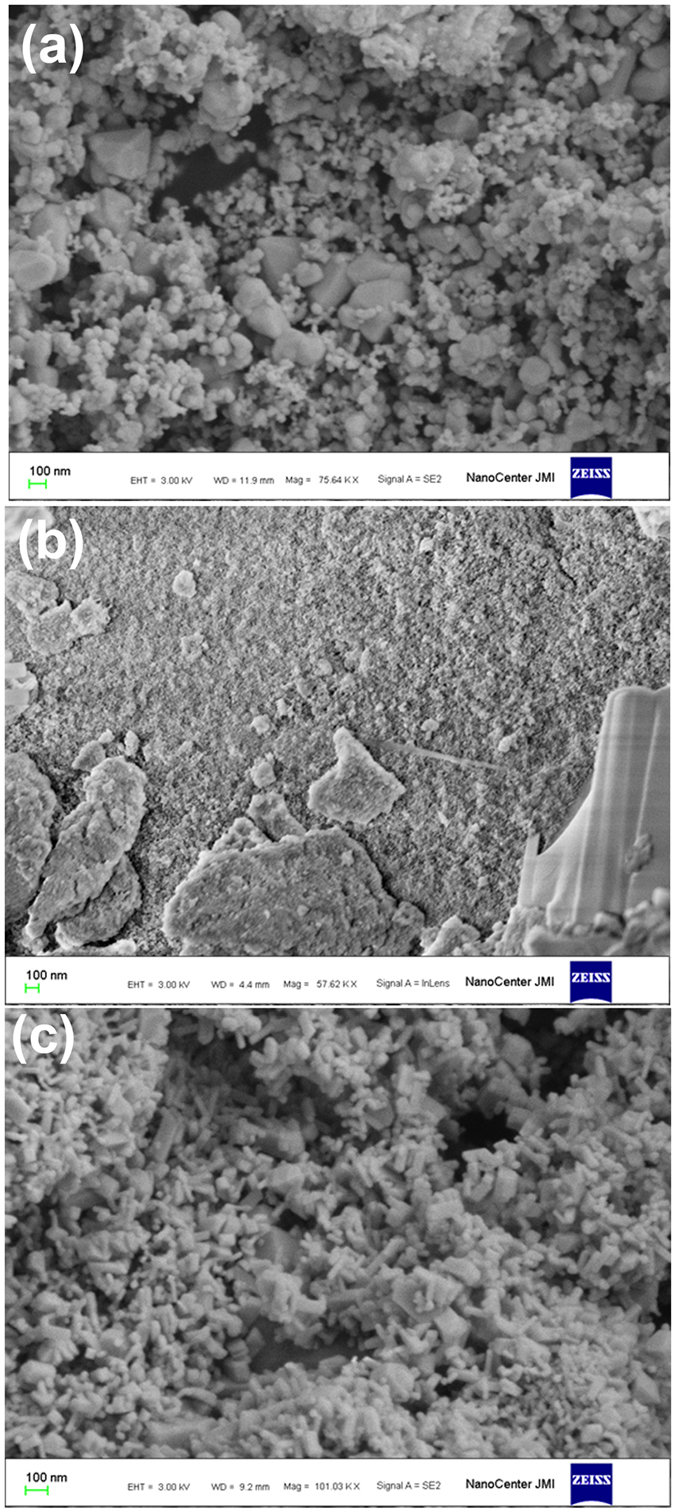
Field emission scanning electron microscopic image of (**a**) pristine SnO_2_ powder, (**b**) Ni-SnO_2_ powder and (**c**) Cr-SnO_2_ powder.

A field emission scanning electron microscopig image (FESEM) of pristine SnO_2_ powder is shown in Fig. 1a, where a mix of spherical and hexagonal particles of approximately 50–150 nm are seen, some of which are slightly agglomerated. The FESEM micrograph of Ni-doped SnO_2_ powder is shown in Fig. 1b, where agglomerated particles forming lumps are seen along with tiny particles that are approximately 30–50 nm in size. The agglomeration seen is a result of calcination of the powder at 1000 °C. Similarly the FESEM image of Cr-doped SnO_2_ powder is shown in Fig. 1c, where rectangular bar/sheet like structure is seen of about 20 nm × 50–100 nm size.

**Figure 2:**
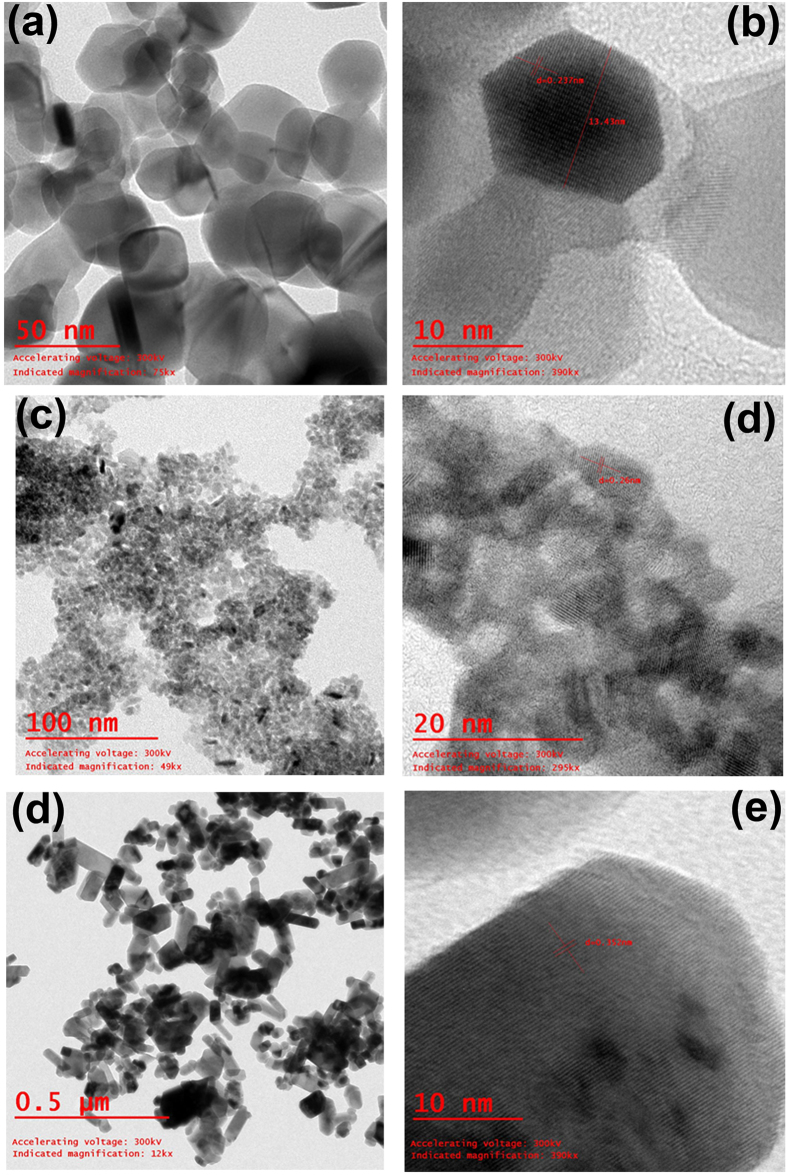
Transmission electron microscopic iamge of (**a**) pristine SnO_2_ powder, (**c**) Ni-SnO_2_ powder and (**e**) Cr-SnO_2_ powder. High resolution TEM image of (**b**) pristine SnO_2_ powder, (**d**) Ni-SnO_2_ powder and (**f**) Cr-SnO_2_ powder.

To further confirm the size and shape, the powders were observed under transmission electron microscope (TEM), and the corresponding TEM and high resolution-TEM (HRTEM) images are shown as Fig. 2(a–f) for pristine, Ni-SnO_2_ and Cr-SnO_2_ powder. In case of pristine SnO_2_ powder (Fig. 2a), the spherical and hexagonal particles are of about 30–80 nm, analogous to FESEM observations. In the HRTEM image (Fig. 2b), the sharp and symmetrical lattices are clearly seen confirming single crystalline nature with a d-spacing of 0.273 nm. Figure 2c shows the TEM iamge of Ni-SnO_2_ powder, where particles of about 20–45 nm are seen, while in the HRTEM image (Fig. 2d), the lattices are sharp and symmetrical with an spacing of 0.260 nm indicating single crystalline phase. The TEM image of Cr-SnO_2_ powder (Fig. 2e) clearly shows a sheet-like structure of approximately 100 nm in length and 20 nm in width. The lattices are clearly seen separated with a distance of 0.152 nm (Fig. 2f).

**Figure 3:**
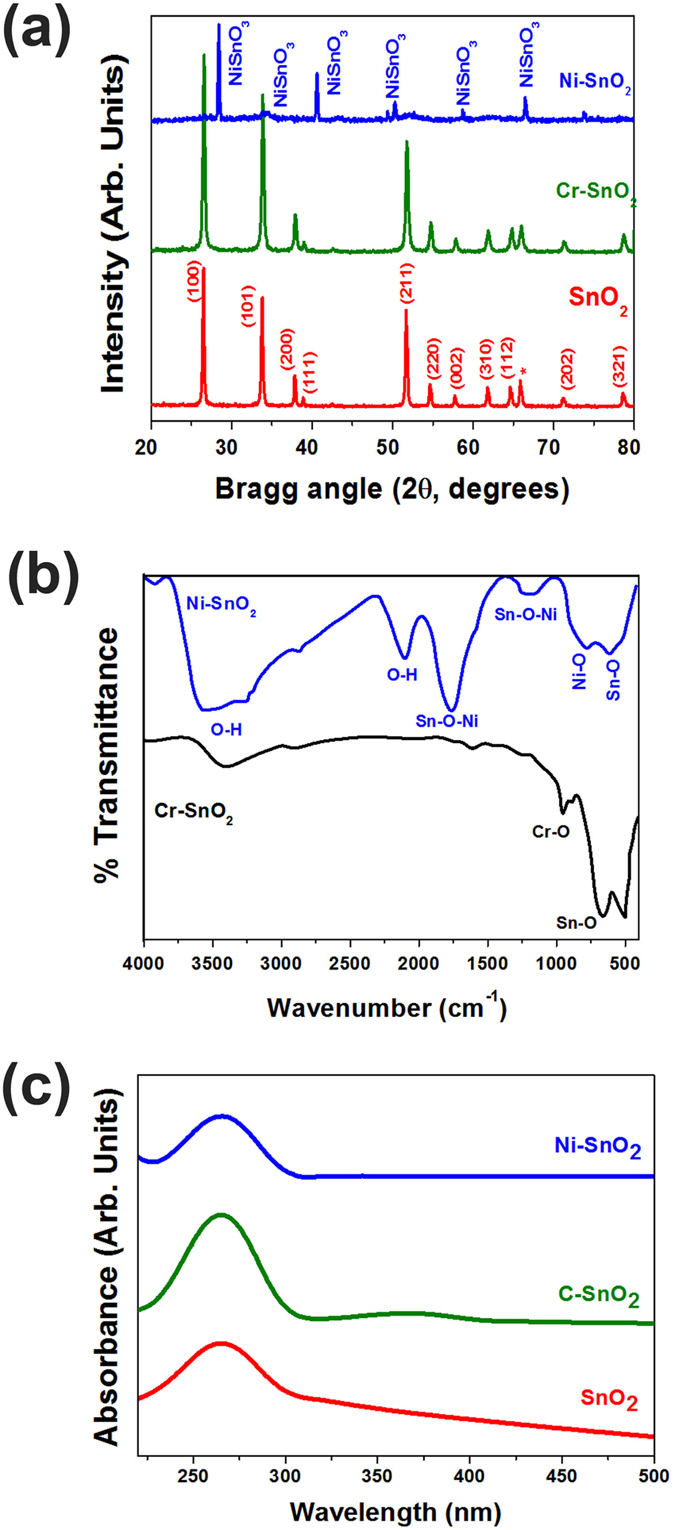
(**a**) X-ray diffraction patterns of pristine SnO_2_ and Ni & Cr-doped SnO_2_ powder, (**b**) FT-IR spectrum of Ni and Cr-doped SnO_2_ powder and (**c**) UV-Visible spectra of pristine SnO_2_ and Ni & Cr-doped SnO_2_ powder.

X-ray diffraction patterns of pristine SnO_2_ and Ni and Cr–doped SnO_2_ powder are shown in Fig. 3a. The spectrum of SnO_2_ matches well with that of standard tetragonal SnO_2_ (JCPDS-41-1445). In the case of Ni-doped SnO_2_, the phases match well with that of the standard NiSnO_3_ phase (JCPDS-28-0711). The sharp diffraction peaks indicate the crystalline nature of the synthesized powder. The average particle size calculated using the Scherrer formula is approximately 82 nm, which is close to that observed from the SEM. In the case of Cr-SnO_2_, the effect of doping could not be detected in XRD, as no additional peak or shift was noticed.

The FTIR spectra of Ni and Cr–doped SnO_2_ is shown in Fig. 3b, where two bands are seen at 674 cm^−1^ and 547 cm^−1^ due to the stretching vibrations of M-O (Ni or Cr) and Sn-O bonds. An asymmetric and symmetric bands due to Sn–O–Ni are observed at 1384 cm^−1^ and 944 cm^−1^ 22, respectively that are not observed in Cr-SnO_2_ powder. The occurrence of bands at 3375 and 1627 cm^−1^ are due to the vibration and deformation frequency of an O-H group because the synthesis was water based.

The UV-Vis spectra of pristine, Ni and Cr–doped SnO_2_ is shown as Fig. 3c, where a symmtetic absorption bands are observed around 264 ± 2 nm for all three samples. The rising band edge is observed around 305 nm, which is similar to the reported data. Similar spectra are observed with Ni ad Cr-doping with slight shift in the peak absorption wavelength.

**Figure 4:**
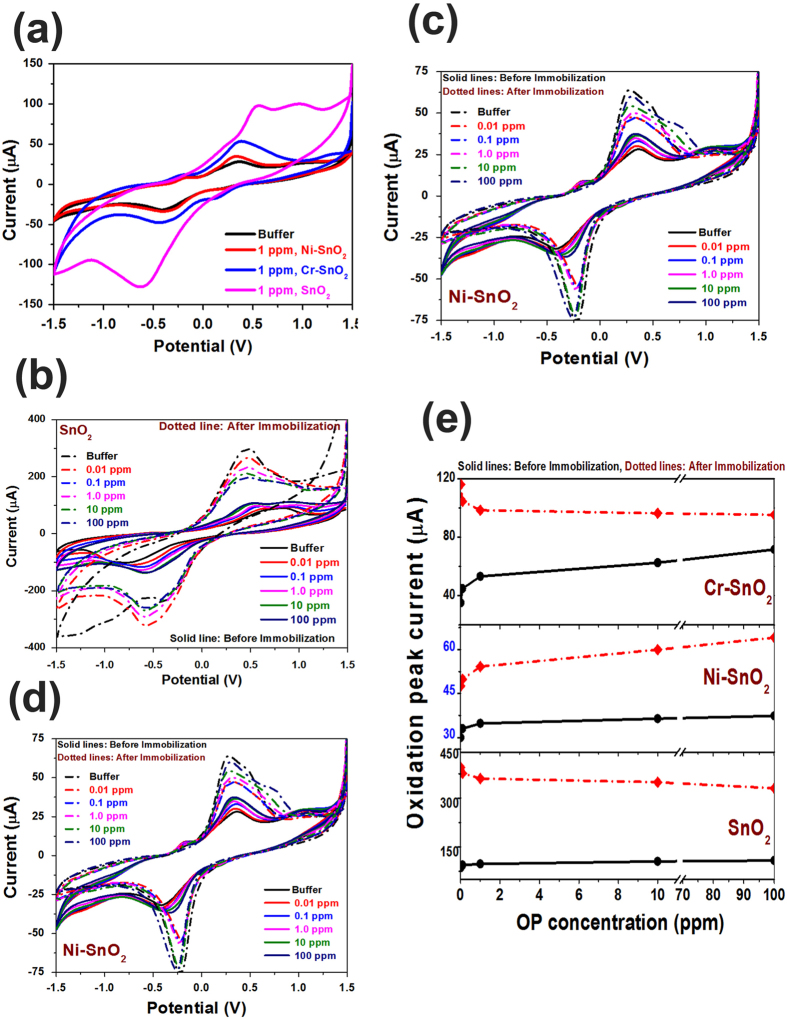
(**a**) variation in CV curves as a function of dopant (Ni and Cr) in reference to pristine SnO_2_, before immobilization in 1 ppm of OP. CV curves as a function of OP concentration before and after AChE immobilization for (**b**) pristine SnO_2_, (**c**) Ni-SnO_2_ and (**d**) Cr-SnO_2_ and (**e**) Variation in the anodic peak current with OP concentration and dopants.

The electrochemical properties of the fabricated sensor were analyzed using a cyclic voltammogram (CV) obtained with a potentiostat. The potential varied from −1.5 to 1.5 V, while the scan rate was kept at 100 mV/S. The CV curves were obtained in the different concentrations of OP solution, shown in Fig. 4. The measurements were performed before and after AChE immobilization at a scan rate of 100 mV/S.

Figure 4a shows the variation in CV curves as a function of dopant (Ni and Cr) in reference to pristine SnO_2_ before immobilization, in 1 ppm of OP. Changes such as loop width, redox peak currents, and peak potential can be clearly seen. In the case of Ni-SnO_2_, redox peaks are seen at 0.34 and −0.38 V, while for Cr-SnO_2_, redox peaks are observed at 0.38 and −0.44 V. In the case of pristine SnO_2_, redox peaks are seen at 0.54 and −0.62 V, with an additional shoulder peak at 0.96 V, indicating the non-stoichiometric nature of SnO_2_. The data clearly show the effect of dopant on the sensing characteristics of the investigated material.

To estimate the sensing characteristics, the CV curves were obtained as a function of OP concentration before and after AChE immobilization, and the curves are shown as Fig. 4b,c and d for pristine, Ni-doped, and Cr-doped SnO_2_, respectively. The OP concentration was varied from 0.01 ppm to 100 ppm. A systematic increase in redox peak currents was noticed with the increasing OP concentration for sensors with and without AChE immobilization. The variation is relatively large when the sensors are immobilized with AChE. Figure 4e shows variation in the anodic peak current with concentration and dopants, where the effect of doping can be seen as changes in the slope, i.e., the sensitivity of the developed sensors. The sensivity of the sensor was estiamated by obtaining the slope of the curves with peak current and OP concentration. The detection limit was estimated following the reported method^15^,^16^.

## Discussion

Table 1 shows a comparison of the redox potential for the pristine and doped material before the immobilization of AChE, with a formal potential value of greater than one, for all of the developed sensors, indicating that the sensors can perform reversibly.

**Figure 5:**
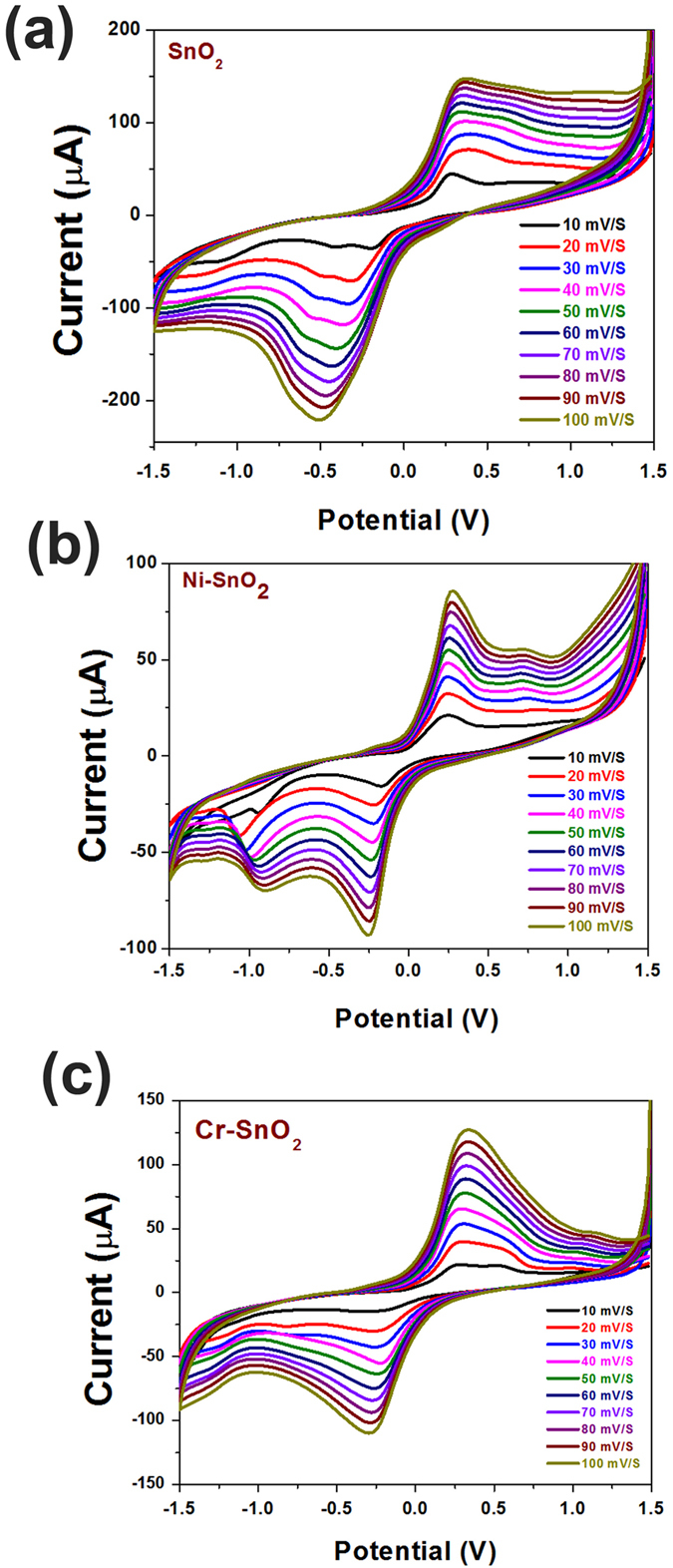
CV curves as a function of scan rate at 1 ppm of OP concentration before AChE immobilization for (**a**) pristine SnO_2_, (**b**) Ni-SnO_2_ and (**c**) Cr-SnO_2_.

**Figure 6:**
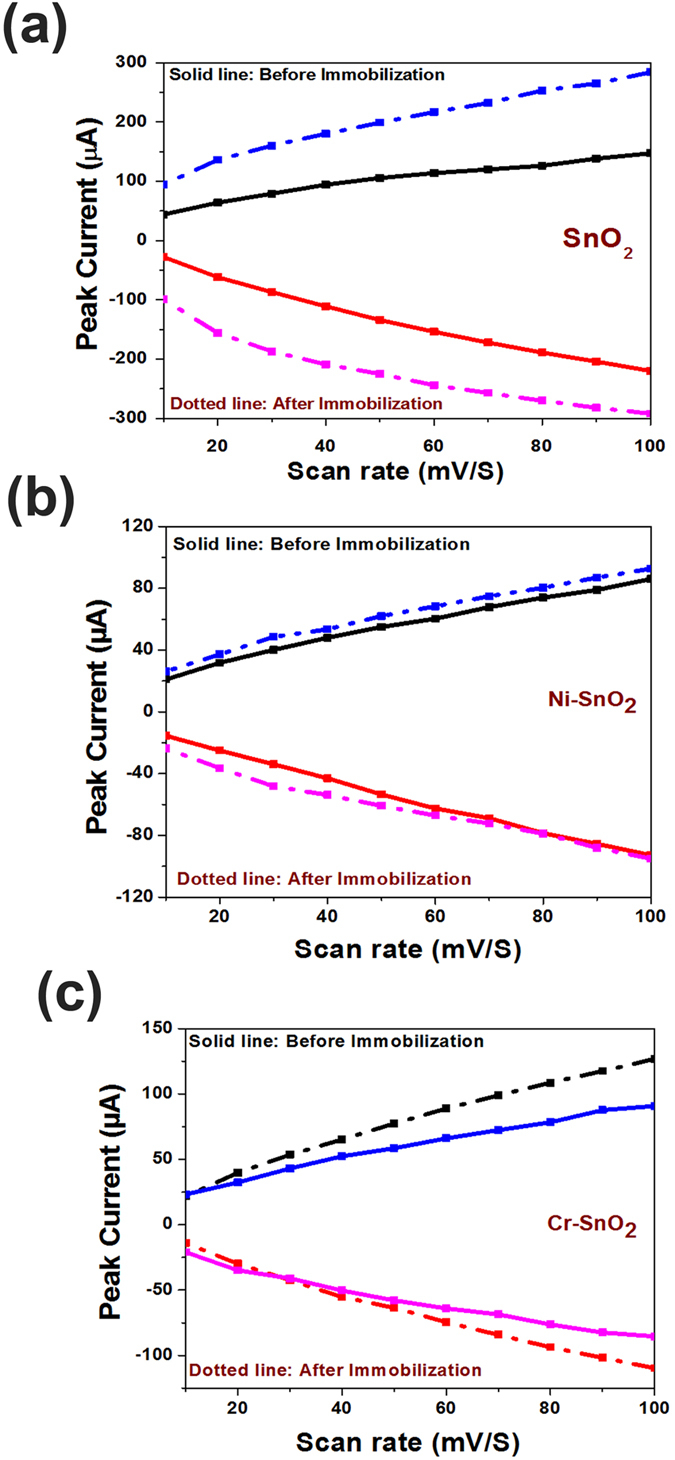
Redox peak current as a function of Scan rate variation for (**a**) SnO_2_, (**b**) Ni-SnO_2_ and (**c**) Cr-SnO_2_.

Furthermore, the scan rate studies were performed before immobilization to understand the effect of prolonged potential exposure on the sensor characteristics and shown as Fig. 5a,b and c for pristine, Ni-doped, and Cr-doped SnO_2_. This study was performed at a fixed OP concentration (1 ppm). A linear increase in the peak current with an increasing scan rate was noted for all of the sensors, indicating linear charge transfer characteristics. SnO_2_ is known to be a good conductor in a given stoichiometric condition, and, hence, it is expected to deliver a linear charge transfer character with increasing potential/time^23^. A similar scan rate variation study was also performed after immobilization; however, data is presented only for the variation in peak current with a scan rate as in Fig. 6a,b and c for pristine, Ni-doped, and Cr-doped SnO_2_.

**Figure 7:**
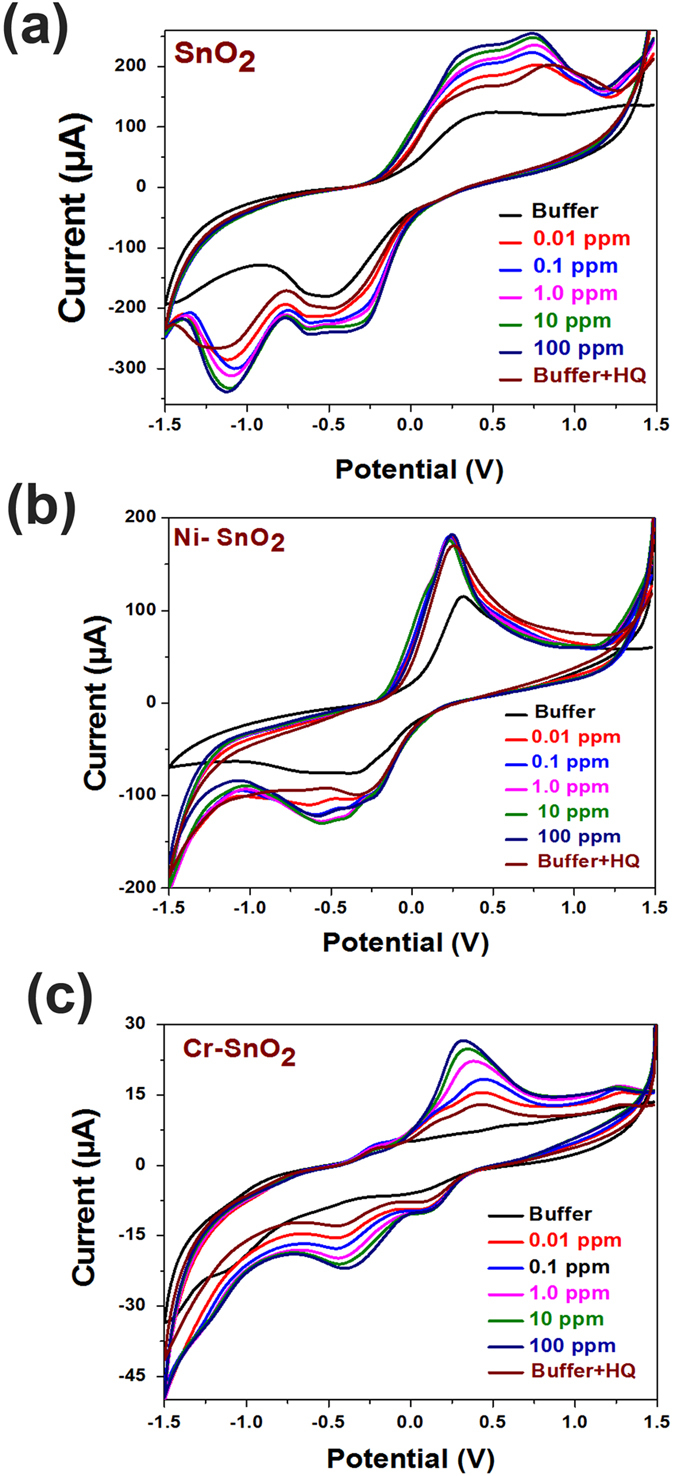
Interference studies with 1 ppm of HQ in various concentrations of OP for (**a**) SnO_2_ (**b**) Ni-SnO_2_ (**c**) Cr-SnO_2_.

To estimate the effect of an interfering agent, an interference study was conducted with 1 ppm of hydroquinone (HQ) mixed in various OP concentrations. Figure 7a–c shows the CV curves of interference studies with SnO_2_, Ni-SnO_2_, and Cr-SnO_2_, respectively. No change in the oxidation peak potential was noticed in any of the samples/powder. In the case of pristine SnO_2_, an additional reduction peak was noticed at approximately −1.12 V. For Ni-doped SnO_2_, an additional reduction peak was noticed at −0.54 V, while for Cr-doped SnO_2_, the reduction peak potential was seen at −0.38 V. It is known that the adsorbed oxygen species will react with HQ other than OP and will result in the further reduction of sensor material by releasing an electron that has resulted as an additional reduction peak. In one of our earlier publications, we presented a detection mechanism based on generation of oxygen species, its reaction with sensor material and role of AChE and OP. The esteratic and anionic subsites of AChE helps in binding OP leading to phosphorylation of AChE. This forms bond between OP and adsorbed oxygen^14^.

A performance comparison was made with the reported sensor, and the parameters estimated from the CV curves are listed in Table 2, which clearly indicates that the synthesized material would be promising for such sensing applications.

## Methods

### Chemicals and Reagent

To study the effect of dopants on the sensing characteristics of SnO_2_, SnO_2_ was doped with Cr and Ni, while commercial grade SnO_2_ (Sigma Aldrich) was used as a reference material. To synthesize doped SnO_2_, anhydrous nickel chloride (NiCl_2_), stannous chloride (SnCl_2_.2H_2_O), and organic solvents were purchased from Sigma-Aldrich. Deionized water (DI, Millipore, 18 MΩ-cm) was used to synthesize this material. The chlorpyrifos (C_9_H_11_C_l3_NO_3_PS) was purchased from Merck in Germany.

### Materials synthesis

The Chimie-douce method was used for material synthesis. In the case of Ni-doped SnO_2_, the typical concentration of chemicals was 11.2 mM of NiCl_2_ and 27.8 mM of KOH mixed in 15 mL of DI water and kept at 80 °C for six hours with continuous stirring, as reported elsewhere^22^. A color change occurred from dark to light green at pH = 10.5. The flask was then cooled to room temperature, followed by the addition of 11.42 mM SnCl_2_ solution. The entire mixture was then refluxed in a three-necked flask for six hours with stirring at 80 °C, and the pH was measured at the same time. When the pH reached to 7.5, the refluxing was stopped, and the solution was filtered after cooling at room temperature. The reaction resulted in a dark green powder that was obtained after several washings with DI water. The synthesized powder was finally calcined at 1000 °C and then used for sensor development.

For Cr-doped SnO_2_, 22.2 mM SnCl_2_.2H_2_O was dissolved in deionized water, followed by the addition of 48 mM KOH at ambient temperature. The reaction was initially exothermic. After cooling, the mixture was refluxed for four hours at 100 °C, resulting in a milky white solution. The pH of the reaction was monitored and was stopped when it reached a value of 7.6; it was then filtered using Whatmann filter paper after several washings with deionized water to remove residual KCl. In the filtrate, 14.9 mM of CrCl_3_.6H_2_O was added after concentrating the filtrate to 20 mL. It was then stirred and refluxed again at 100 °C for 4 hours and was filtered afterwards using Whatmann filter paper. The nanopowder was separated out as a grey precipitate after several washings with deionized water and was finally calcined at 1000 °C to be used for sensor development.

In this report, an electrochemical sensor was fabricated using pristine, Ni-doped, and Cr-doped SnO_2_ as a tool to detect agro-hazardous material, i.e. OP (chlorpyrifos). The Ni and Cr–doped SnO_2_ nanomaterial was synthesized using the solution method and used as a matrix after it was calcined at 1000 °C. Morphological observations confirmed the nanosize of the synthesized material and could therefore be a better matrix material, as it offers a higher adsorption area. All of the material showed a response to the OP, but Ni-doped SnO_2_ showed better sensing characteristics. This study indicated the possibility of using such a material matrix for developing a device that can serve the needs of the food and agro-industries for the quick and reliable detection of agro-hazardous materials.

Electrochemical sensing properties were studied using a potentiostat/galvanostat (Ivium, the Netherlands) by obtaining CV and scan rate studies. The potential was cycled from −1.5 to +1.5 V at a scan rate of 100 mV/S. To understand charge transfer properties, scan rate variation was studied. An OP solution was prepared in PBS (pH 7.2, 0.1 M) to be used as an electrolyte. A OP (pesticide) solution of different concentrations (0.01, 0.1, 1, 10, 100 ppm) was prepared in PBS. These characteristics were studied with and without AChE (10 μL from a 5 mg/250 mL solution). Different scan rate studies of the particular analyte at a concentration were performed using a potentiostat (Ivium). Scan rates were varied from 10, 20, 30, 40, 50, 60, 70, 80, 90, to 100 mV/S, and the potential was cycled from −1.5 to +1.5 V. The scan rates dependent characteristics were obtained with and without AChE enzyme immobilization. An interference study was conducted by mixing 1 ppm of hydroquinone (HQ) in various OP concentrations.

### Instrumentation

The morphology of the powder was observed under a field emission scanning electron microscope (FESEM, Supernova, ZEISS) and transmission electron microscope (TEM), High resolution (HRTEM, 300 KV, Tecnai, FEI), while an X-ray diffraction pattern (Ultima IV, Rigaku) with a CuKα target (1.5415 Å) was obtained to ascertain the structural information. Functional band information was observed with a Fourier transform infrared (FTIR) spectrophotometer (Tensor 37, Bruker) in attenuated total reflection (ATR) mode.

## Additional Information

**How to cite this article:** Khan, N. *et al*. Application of pristine and doped SnO_2_ nanoparticles as a matrix for agro-hazardous material (organophosphate) detection. *Sci. Rep.* **7**, 42510; doi: 10.1038/srep42510 (2017).

**Publisher's note:** Springer Nature remains neutral with regard to jurisdictional claims in published maps and institutional affiliations.

## Figures and Tables

**Table 1:** Oxidation/Reduction peak potential and formal potential values.

	SnO_2_	Ni-SnO_2_	Cr-SnO_2_
Oxidation Potential (V)	0.54	0.34	0.38
Reduction Potential (V)	−0.62	−0.38	−0.44
Formal Potential (V)	1.08	1.04	1.06

**Table 2:** Comparison of the analytical methods for the detection of OP.

Analytical methods	Sensitivity (μA/ppm)	Detection limit (ppm)	References
AChE/ MWCNTs-TCNQ/SPE	—	0.1	24
AChE/CPBA/GR-AuNPs/GCE	—	0.1	25
BSA/anti-chlorpyrifos/GSMB/GNPs/GCE	—	0.056	26
AChE/Au/PB/GCE	—	2.0 × 10^−2^	27
AChE/Nf/TCNQ/SPCE	—	2.1	28
ACHE/Au-Fe_3_O_4_/GCE	—	8.6 × 10^−2^	29
AChE/ZnO/SPE	0.01	0.31	30
AChE/PrZnO(1.1)/SPE	0.1	0.17	30
AChE/PrZnO(1.10)/SPE	0.011	0.24	30
AChE/SnO_2_/SPE	4.3 × 10^−2^	0.51	Present work
AChE/Ni-SnO_2_/SPE	1.23 × 10^−2^	0.18	Present work
AChE/Cr-SnO_2_/SPE	0.9	0.98	Present work
